# Home-based exercise using balance disc and smartphone inclinometer application improves balance and activity of daily living in individuals with stroke: A randomized controlled trial

**DOI:** 10.1371/journal.pone.0277870

**Published:** 2022-11-21

**Authors:** Pantawit Aphiphaksakul, Akkradate Siriphorn

**Affiliations:** Department of Physical Therapy, Human Movement Performance Enhancement Research Unit, Faculty of Allied Health Sciences, Chulalongkorn University, Bangkok, Thailand; Duta Wacana Christian University School of Medicine / Bethesda Hospital, INDONESIA

## Abstract

**Background:**

Sitting ability is critical for daily activities in individuals who have experienced a stroke. A combination of seated balance training on an unstable surface and real-time visual feedback via a simple mobile inclinometer application may improve trunk control in stroke survivors.

**Objective:**

This randomized controlled trial aimed to determine the effects of home-based exercise utilizing a balance disc with input from a smartphone inclinometer application on sitting balance and activities of daily living in stroke survivors.

**Methods:**

This trial enrolled 32 stroke survivors aged 30 to 75 years. Participants were randomly assigned to one of two groups: intervention or control. Both groups underwent four weeks of traditional therapy. Additionally, the intervention group received four weeks of multidirectional lean training utilizing a balance disc and a smartphone application with an inclinometer. The Postural Assessment Scale for Stroke (PASS), the Function in Sitting Test (FIST), and the Barthel Index (BI) were used to assess the results. To compare between group effects, an ANCOVA analysis was performed using a baseline as a covariate.

**Results:**

The PASS changing posture and BI were considerably greater in the intervention group compared to the control group. Other metrics revealed no statistically significant differences between the groups.

**Conclusion:**

Home-based training with balance discs and input from a smartphone inclinometer application may improve postural control and daily activity in stroke patients.

**Trial registration:**

Clinical trials registry number: TCTR20210617004.

## Introduction

Most cases of stroke result in long-term disability due to impairment of many processes in the body, including muscle weakness, deprivation of proprioception, and loss of feeling [1, 2]. Strokes affect physical functions such as sitting, standing, and walking, and restricts other physical activities [3]. Difficult with mobility and physical activity hinders the lifestyle of stroke survivors and often leads them to remain bedbound [4].

Stroke rehabilitation is a critical component of stroke treatment because it increases brain activation and mobility through physical activity. The rate of recovery tends to be rapid in the first three months and continues to improve until six months. This time period is critical for training and increasing everyday activity levels [5]. Thus, people who have had a stroke should exercise to improve their strength, cognitive abilities, balance, mobility, and balance soon after suffering the stroke [6]. Stroke rehabilitation can begin immediately after the patient has been medically stabilized in intensive care environments [7]. Previously, the timing and intensity of rehabilitation training for stroke survivors were limited in a hospital setting, resulting in inadequate recovery. It was later advocated that all stroke patients undergo rehabilitation therapy in inpatient facilities for three hours per day with rigorous task-specific training five days per week for three weeks [7]. Following hospital discharge, a significant number of stroke patients may require continued outpatient therapy and additional long-term community rehabilitation for several months or years [6, 8, 9]. Numerous community-based rehabilitation approaches, including home-based virtual reality training [10], robotic gloves [11], and telerehabilitation [12], have been examined. However, the majority of home-based rehabilitation programs rely on expensive technology and are primarily focused on rehabilitating hand functions [1, 9, 13, 14].

Sitting is an important part of everyday activities, as well as the transition to standing and performing other movement [15]. Stroke patients who previously struggled to maintain their sitting balance have slow progress in transitioning to standing, which impairs walking rehabilitation [16]. Trunk control training has been shown to improve the spatiotemporal gait parameter in individuals with chronic stroke and can help improve sitting balance [16–18]. Various trunk control exercises are appropriate for stroke survivors, including static sitting balance without back or arm support, dynamic sitting balance on a stable surface, and dynamic training balance on an unstable surface using a physio ball [17, 18]. Such trunk balancing exercises improved balance, functional state, and ambulation more than conventional stroke exercises [19]. A recent meta-analysis showed that using trunk-based inpatient rehabilitation techniques improved short-term trunk efficiency and balance in stroke survivors [20]. However, these exercises were conducted while stroke patients were hospitalized, which may have been insufficient to enhance long-term sitting balance and other functional abilities. Chan et al. (2015) conducted the only study that examined task-related trunk training as a home-based program for stroke survivors. The six-week program included five training sessions each week. Each training session consisted of six sets of exercises, including pelvic bridging, supine-to-sit, forward-backward and lateral leaning, trunk rotation, and forward reaching while sitting. The findings indicated that stroke patients completing the training program had greater increase in trunk muscle strength and function [21].

Visual feedback is another important factor of balance training’s effectiveness. Cho et al. (2012) demonstrated that six weeks of visual feedback via video game-based training for three days per week, 30 minutes per day enhanced dynamic balance in stroke survivors [14]. Similarly, Karasu et al. (2018) demonstrated that when used in conjunction with other treatments, the Nintendo Wii system led to improved static and dynamic balance in individuals with stroke compared to standard treatments without the visual feedback aspect [22]. Interestingly, Hwang et al. (2017) demonstrated that a 30-minute session of visual feedback training utilizing a force platform three days per week for four weeks was more effective than unstable surface training at enhancing static and dynamic balance in stroke survivors [23]. Thus, we hypothesized in this study that combining sitting balance training on an unstable surface with real-time visual feedback through a simple mobile inclinometer application would improve stroke survivors’ trunk control following discharge. The purpose of this study was to determine the impact of home-based training utilizing a balance disc with input from a smartphone inclinometer application on sitting balance and daily living activities in individuals recovering from stroke.

## Material and methods

### Research design

The study employed a single-blind, parallel-group randomized controlled design. The researcher conducted a screening of stroke survivors who volunteered to participate in the study. All individuals who met the inclusion criteria for screening were examined for demographic data. This study employed stratified randomization, in which participants were randomly assigned based on their age, gender, and body mass index. The participants were randomly assigned to the intervention or control groups using a concealed opaque envelope with a computer generated random number. These concealed opaque envelopes were not opened until all screening and baseline outcome measures were completed. A research assistant who was not involved in outcome assessments or treatments was responsible for participant randomization. The intervention group received a 30-minute sitting balance training program for five days per week for four weeks in addition to a 30-minute conventional home rehabilitation program. The control group received 60 minutes of conventional rehabilitation treatment five days per week for four weeks. All outcomes were assessed twice at home; once one week after discharge (pre-test) and once at the completion of the training session (post-test). This trial was registered before enrollment of the first participant with the Thai Clinical Trials Registry (www.thaiclinicaltrials.org), number TCTR20210617004. This study was approved by the Research Ethics Review Committee for Research Involving Human Research Participants, Health Sciences Group, Chulalongkorn University (COA. 049/2021). Before taking part in this study, all participants signed written informed consent forms. The individual pictured in Fig 2 has provided written informed consent to publish their image alongside the manuscript.

### Sample size

The sample size was calculated using version 3.1.9.7 of the G*Power program based on the expected effect size of 0.50. The result of the sample size estimation when setting power at 80%, alpha at 0.05, was 26 participants (13 per group). The drop-out rate was set at 20%, requiring a minimum of 32 participants (16 per group).

### Participants

**Fig 1:**
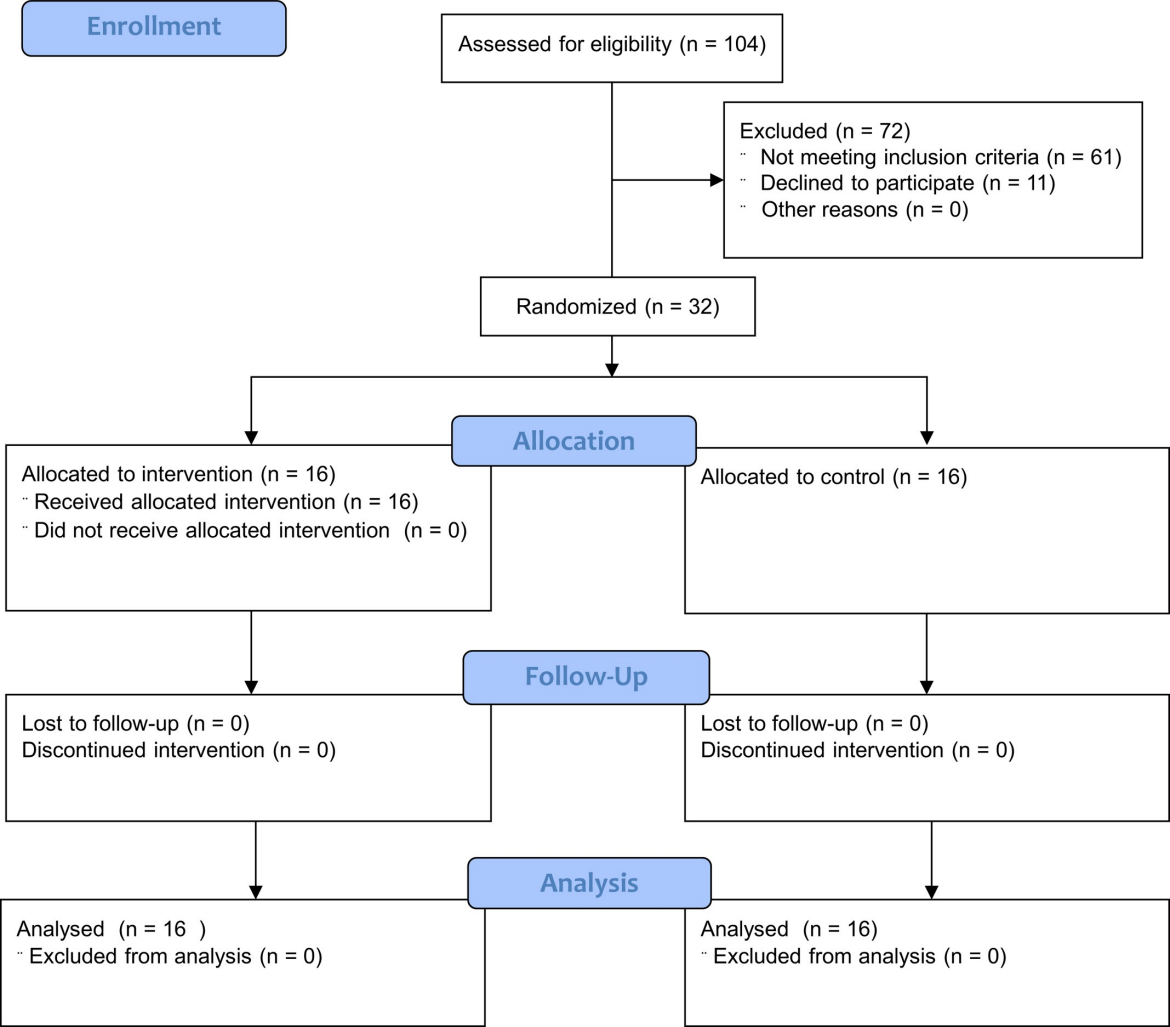
CONSORT flow chart diagram for enrollment, allocation, follow-up, and data analysis.

Between August 2021 and February 2022, 32 stroke survivors aged 30 to 75 years were recruited from the Bangkok metropolitan region by direct contact with physical therapists who cared for stroke patients at home. Fig 1 depicts the study’s participant CONSORT flow diagram. All participants were informed about the testing and training procedures. Before participating in this study, all subjects provided informed consent. The following criteria were used to enroll participants: 1) diagnosis of the first stroke resulting in hemiplegia within the preceding three months; 2) release from the hospital and return to home care; 3) aged between 30 and 75 years; 4) modified Rankin scale score of 3 or more; 5) ability to sit independently without assistance; 6) Postural Assessment Stroke patient (PASS) score of 12.5 points or less; 7) family members or caregivers were able to assist with instrument setup and ensure patient safety during the home-based intervention; 8) no vision problems that cannot be corrected with glasses or contact lenses; 9) no history of back surgery, scoliosis, and no current low back pain that impairs sitting performance; 10) capable of communicating and following instructions. The following criteria were used to exclude participants: 1) diagnosis of another neurological issue, such as Parkinson’s disease or cerebellar disorder; 2) uncontrolled hypertension; 3) unstable sign or accident during the trial; and 4) inability to obey an instruction.

### Research protocol

All participants were screened using a questionnaire to ascertain participant characteristics and an eligibility checklist to determine their eligibility based on inclusion and exclusion criteria. The screening procedure was performed at the participants’ homes and was conducted by a research assistant who was not involved in the intervention. The researcher, who was not engaged in the outcome assessments, gave participants an exercise routine at their homes, which consisted of a leaflet outlining the workout prescription. The family members or caregiver received comprehensive training on how to administer the exercise intervention with the participants. A balance disc, a chest strap with an extended arm, a smartphone, and a chair were distributed to the intervention group.

**Fig 2:**
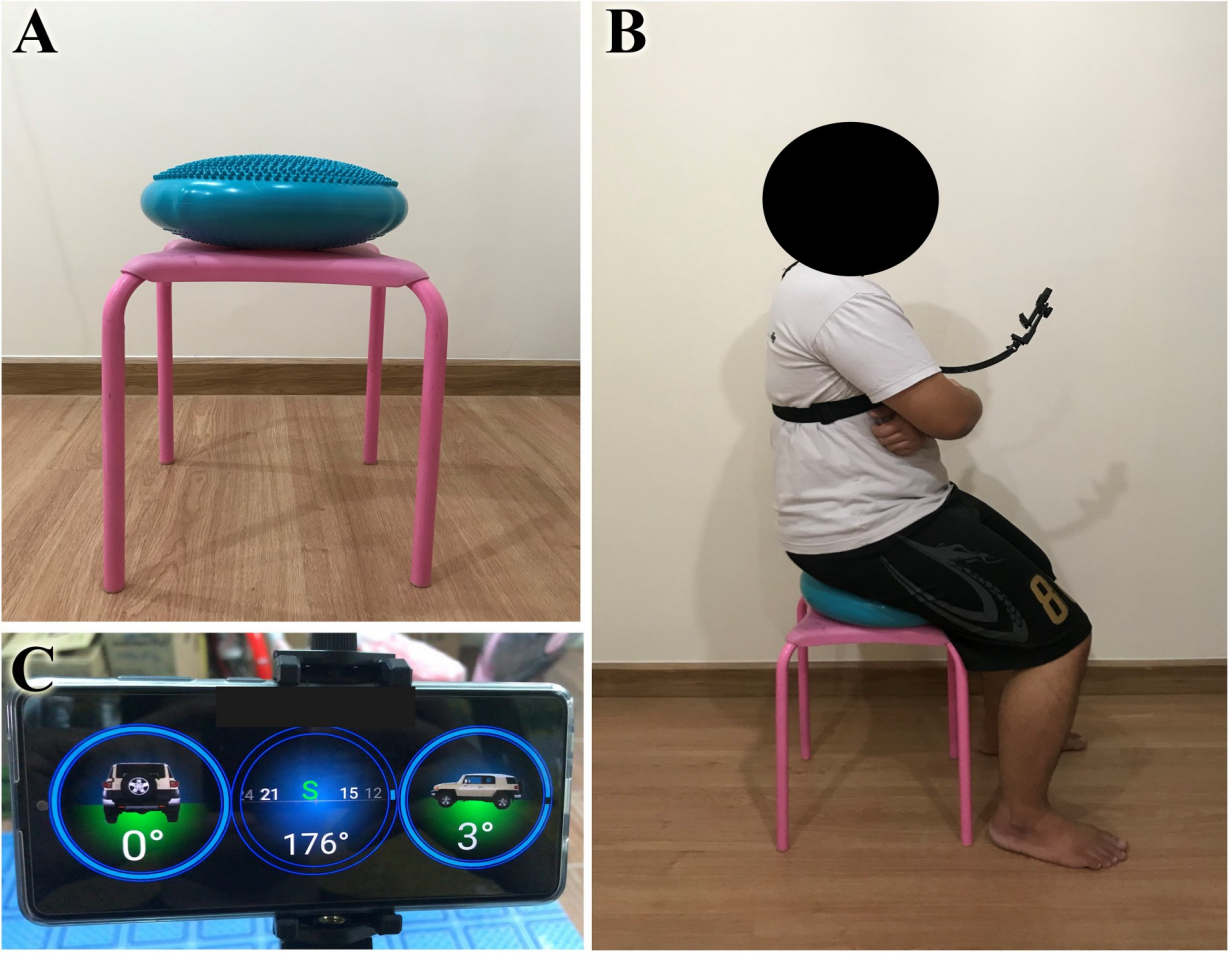
Setup of a home-based exercise using balance disc and smartphone inclinometer application. (A) A balance disc and chair. (B) Starting position. A chest strap and an extension arm were used to secure the smartphone to the participants’ chests. (C) The Compass Inclinometer application.

The smartphone was pre-installed with the Compass Inclinometer application, which is a free downloadable Android OS application (Brain Laboratories, accessible at https://play.google.com/store/apps/details?id=com.brainlab.tiltmeter). The researcher demonstrated operation of the equipment and training program. The smartphone was placed on the participants’ chests using a chest harness and an extension arm was attached to the smartphone (Fig 2). On the screen, the program displayed three inclinometers, each of which indicated the degree of tilt of the smartphone. During exercise, participants were to look at the smartphone screen and move according to the exercise program.

To begin, the participant sat on a balance disc put on a chair with their feet flat on the floor. The researcher employed a step platform to support both feet if the participant’s feet could not be flat on the floor. Participants were obliged to wear a belt around their waist at all times. The researcher educated family members or caregivers to ensure the participant’s safety during the exercise by standing behind the participant and placing their hand near the belt. The exercise program consisted of five levels which were modified from a previous study [24]:

Level 1: Two minutes of static sitting with no movement or less than a five-degree shift in tilt observed by the inclinometer.

Level 2: Lean forward, backward, lateral, and rotate the trunk to both sides using an inclinometer set to 15 degrees. This consisted of moving to the end position, holding for five seconds, then returning to the beginning position. This was repeated 15 times per set for three sets of each direction.

Level 3: Same as level 2, but with an inclinometer set to 30°.

Level 4: Same as level 2, but with an inclinometer set to 45°.

Level 5: Same as level 2, but with an inclinometer set to 60°.

Each participant began at level 1. Participants could advance to the next level if they completed each level successfully in three consecutive sessions. Family members or caregivers were encouraged to evaluate whether the participant passed or failed the level. If participants failed the level, they restarted training at the previous level until they accomplished that level correctly and completely. The intervention group participated in the 30-minute sitting balance program five days per week for four weeks, in addition to the 30-minute conventional home rehabilitation program. The family members or caregiver was requested to provide support for the home-based program, specifically for participants’ safety concerns during the exercise program. The control group was instructed to undergo the 60-minute conventional home rehabilitation program five days per week for the four-week study period. This program included basic physical movements such as passive and active range of motion exercises on both sides of the body, bed mobility training, and seated balance training with supervision or assistance from a family member or caregiver.

### Outcome measurements

All evaluations were carried out on the basis of pre- and post-testing data collected from each participant. All of the outcome measurements were done by a research assistant who was a certified physical therapist and was blinded to participants’ group assignment. The function in sitting test (FIST), the postural assessment scale for stroke patients (PASS), and the Barthel Index (BI) were used as outcome measures.

#### Function in sitting test (FIST)

The FIST was used to measure clinical balance. This test assesses the performance in sitting balance based on the following 14 items: 1. Anterior nudge, 2. Posterior nudge, 3. Lateral nudge, 4. Static sitting, 5. Sitting, moving head side to side (nod “no”), 6. Sitting, eyes closed, 7. Sitting, lifting the foot, 8. Turning and picking up objects from behind in preferred direction, 9. Reaching forward with outstretched hand at shoulder height, 10. Lateral reaching with a hand at shoulder height, 11. Picking object up off floor, 12. Posterior scooting (5 cm.), 13. Anterior scooting (5 cm.), and 14. Lateral scooting (5 cm.). Each item is scored on a five-point scale (0–4), with lower scores indicating lower ability to complete tasks without assistance. The maximum FIST score is 56 [25] and the minimal clinically important difference (MCID) of FIST in stroke is 3.5 points [26].

#### Postural assessment scale in stroke patients (PASS)

The PASS is used as a clinical balance measurement tool. The PASS consists of the following 12 items: 1. sitting without support, 2. standing with support, 3. standing without support, 4. standing on a non-paretic leg, 5. standing on a paretic leg, 6. supine to affected side lateral, 7. supine to non-affected side lateral, 8. supine to sitting up on the edge of the mat, 9. sitting on the edge of the mat to supine, 10. sitting to standing up, 11. standing up to sitting down, 12. standing, picking up a pencil from the floor) to evaluate posture change position(dynamic). A four-point scale ranging from 0–3 points is used (zero indicates the lowest level of function, and three indicates the highest level of function). The maximum PASS score is 36. Previous research found a cut-off score of 3.5 for PASS maintaining posture, 8.5 for PASS changing posture, and 12.5 for total PASS accurately predicted stroke patients’ capacity to walk independently after discharge [27].

#### Barthel index (BI)

The BI is used to quantify daily life activities. It includes the following ten activities of daily living: 1. feeding; 2. bathing; 3. grooming; 4. dressing; 5. bowels; 6. bladder; 7. toilet usage; 8. transfer (bed to chair and back); 9. mobility (on level surfaces); and 10. stairs. This tool is rated on a three-point scale: zero indicates that the task cannot be completed even with assistance, five indicates that it requires assistance, and ten indicates that it is autonomous. The maximum BI value is 100 and the MCID of BI in stroke is 1.85 points [28].

### Statistical analysis

The data were analyzed using SPSS version 20.0 for statistical analysis, with a significance level set at p = 0.05. For the demographic and clinical data, descriptive statistics were employed. Unless otherwise specified, all data were provided as mean standard deviation. To determine the normal distribution of the data, the Shapiro-Wilk test was utilized. The pair *t*-test was used to examine within-group differences at the pre- and post-test. According to the research design, which was a two-arm randomized pre-post design, Analysis of Covariance (ANCOVA) was used to identify between-group effects using pre-test score as a covariate, as it was found to be the simplest and most efficient method for analyzing the results of this research design [29].

## Results

This research enrolled 32 participants, including 13 males and 19 females. The four-week program was completed by all participants without any discontinuations. Fig 2 illustrates the participants’ flow diagram. Table 1 summarizes the demographic characteristics of the participants. A comparison of the general characteristics of participants in the intervention and control groups yielded no significant differences. The majority of participants (87.5%) had experienced ischemic strokes. All participants had a moderately severe disability, as measured by a Modified Rankin Scale score of 4. Participants of the study reported no adverse effects. All participants in the intervention group completed more than 80% of the exercise regimen (4 participants completed all 20 sessions, 6 participants completed 17 sessions, and 6 participants completed 16 sessions). Hence, the average rate of exercise adherence was 87%. After completing the training, 12 individuals (75%) achieved Level 3, only four participants (25%) achieved Level 4, and none of the participants achieved Level 5 of the exercise regimen.

**Table 1:** Characteristics of participants in the intervention and control groups.

Variable	Intervention	Control	*p*-value
	(n = 16)	(n = 16)	
**Age—**y; mean (SD)	59.38 (11.09)	59.38 (10.80)	1.000 ^a^
**Sex**—female/male; n	10/6	9/7	0.723 ^b^
**Weight**—kg; mean (SD)	53.31 (9.63)	53.88 (7.68)	0.856 ^a^
**Height**—cm; mean (SD)	159.13 (8.57)	158.50 (8.38)	0.836 ^a^
**BMI**—kg/m^2^; mean (SD)	21.07 (3.30)	21.40 (2.24)	0.747 ^a^
**Time from onset of stroke to assessment**—day; mean (SD)	45.63 (18.91)	37.94 (19.09)	0.262 ^a^
**Type of stroke**—ischemic/hemorrhagic; n	15/1	13/3	0.293 ^b^
**Side of hemiplegia**—right/left; n	7/9	11/5	0.161 ^b^
**Modified Rankin Scale**—mRS score 4 (moderately severe disability); n	16	16	-

^a^ *t*-test

^b^ Chi-squared test.

Table 2 shows the mean and results for each outcome measurement. Participants in both the intervention and control groups significantly improved their FIST, PASS total score, PASS sub-scores, and BI at post-test. ANCOVA analysis with pre-test as covariate revealed that the intervention group scored significantly higher on the PASS changing posture sub-score (adjusted mean group difference of 1.69, F (1, 29) = 7.51, *p*-value = 0.010, partial eta squared of 0.21, and observed power of 0.76) and BI (adjusted mean group difference of 5.92, F (1, 29) = 6.25, *p*-value = 0.018). The BI group difference was clinically significant because it exceeded the minimum clinically important difference (MCID) of the BI in stroke patients, which was 1.85 points, and the observed power was a large difference effect. The MCID of PASS score change also met the threshold for a large difference effect; however, no research had yet demonstrated the MCID of PASS changing score. No statistically significant difference in any other parameter was observed between groups (Table 2).

**Table 2:** Function in sitting test (FIST), postural assessment scale in stroke patients (PASS; total, maintaining posture, changing posture), and Barthel index (BI) at pre- and post-test of intervention and control groups.

		Group		Between group				
		Intervention	Control	AMD	F	*p*- value ^b^	Partial eta^2^	Power
		(n = 16)	(n = 16)					
		Mean (SD)	Mean (SD)					
**FIST**	Pre	27.56 (4.53)	22.75 (4.27)					
	Post	42.06 (2.77)	37.44 (6.31)	**1.31**	0.69	0.41	0.02	0.**1**3
Within group *p*-value ^a^		**<0.001** *	**<0.001** *					
**PASS total**	Pre	9.81 (1.72)	7.81 (2.37)					
	Post	22.31 (2.70)	17.75 (5.18)	**1.50**	1.99	0.169	0.06	0.28
Within group *p*-value ^a^		**<0.001** *	**<0.001** *					
***PASS maintaining posture***	Pre	4.06 (0.68)	3.25 (0.93)					
	Post	9.13 (1.41)	7.56 (2.22)	**0.68**	1.06	0.312	0.04	**0.1**7
Within group *p*-value ^a^		**<0.001** *	**<0.001** *					
***PASS changing posture***	Pre	5.75 (1.65)	4.56 (1.82)					
	Post	13.19 (1.72)	10.19 (3.10)	1.69	7.51	0.010*	0.21	**0.**76
Within group *p*-value ^a^		**<0.001** *	**<0.001** *					
**BI**	Pre	32.19 (7.52)	26.88 (9.81)					
	Post	62.19 (6.05)	53.44 (9.26)	5.**92**	6.25	0.018*	0.18	**0.6**8
Within group *p*-value ^a^		**<0.001** *	**<0.001** *					

* Significant difference

^a^ Within group p-value was calculated by pair t-test

^b^ Between group p-value was calculated by ANCOVA with pre-test as covariate; AMD: Adjusted mean group difference; PASS: Postural Assessment Scale for Stroke; FIST: Function in Sitting Test; BI: Barthel Index.

## Discussion

The purpose of this study was to determine the effect of home-based balance training using an inclinometer smartphone application on stroke patients’ trunk control capacity and daily activity. After four weeks, both the intervention and control groups improved on all outcome measures, with the intervention group significantly outperforming the control group on the PASS changing posture sub-score and BI score. This intervention emphasized leaning training while seated on a balancing disc and receiving feedback from an inclinometer application. The improvement observed in the intervention group suggests that this home-based intervention might well be utilized to enhance trunk control in individuals recovering from stroke.

Stimulation and training are critical during the first three months following a stroke to regain efficiency at balancing tasks, muscular power, and mobility [17, 30]. One of the early rehabilitation strategies is transitioning from a lying to a sitting posture, which has been shown to be beneficial in stroke treatment. Therefore, proper sitting balance is crucial during this time, as it may influence a stroke patient’s ability to perform a number of daily functions and activities [31].

A previous study found that a 30-minute stroke physical therapy program combined with an extra 30-minute balance training program every day for four days per week for five weeks enhanced balance ability in individuals who had suffered a stroke [32]. A 30-minute conventional physical therapy program combined with 30-minute balance training was employed in this study for five days per week for four weeks, for a total of ten hours of balance training as in the prior study.

In this study, participants in the intervention group used an inclinometer application on their smartphones and a balance disc to adjust or maintain body positions by moving their trunk and pelvis in a variety of directions. The movements included forward and backward leaning, lateral trunk bending to both the affected and less-affected sides, and left and right rotation. This intervention technique may have contributed to the intervention group’s enhanced trunk control abilities, as participants received real-time feedback on their mobility in each direction via the inclinometer smartphone application [33]. As individuals adjusted their body based on the feedback, they become more competent in planning and preparing subsequent motions [34]. This is a critical part of movement management because it keeps movement smooth and balanced. Thus, the intervention in this study may have an effect on numerous components of the learning process, including movement preparation and planning, body and lower limb coordination, and sitting motion control [35, 36]. As an additional challenge for participants, the target degrees of leaning in each direction were gradually increased in accordance with the participants’ capacity to surpass their previous best performance. This type of training strategy might result in increased learning and attention, as well as a sustained improvement in sitting balance [37].

The capacity to balance in a sitting position while changing posture was substantially different across groups after training, although maintaining posture while sitting was not significantly different. These results might be explained by the concept of training specificity, since the training in this study taught patients to lean in different directions thereby allowing for the improvement of dynamic balance measures but not static balance measurements.

In this study, participants exercised at home, which may be advantageous compared to other training locations and is facilitated by today’s readily available smartphones [38]. Using smartphones as the basis for a patient training regimen is beneficial because it facilitates on-demand exercise guidance and continuation of patient training after discharge [38, 39]. A simple-to-use, programmable tool that offers feedback on the degree and direction of movement should be utilized in conjunction with equipment that can be configured for home use [40]. While certain commercial gaming systems, such as the Nintendo Wii or Kinect [10, 41], have been harnessed to offer visual feedback to stroke patients, access to equipment and the use of video games to train patients may be limited due to cost [42]. In contrast, most participants already owned a smartphone upon entering the study. Thus, use of the smartphone as a feedback device in conjunction with a low-cost balance disc improves training accessibility [40]. Furthermore, the smartphone applications that were employed in the study were free to participants and easy to use [43]. Using smartphones for visual feedback training, this study successfully demonstrated the positive impact of visual feedback training on the capacity of stroke patients to control their own bodies, which can aid improvement in their trunk control and overall quality of life [6, 44].

The primary strengths of this research are that it was one of the first to employ smartphones and other commonly available devices for balance training in stroke patients, and that it incorporated simple home-based exercise [21, 33]. The outcomes of this study were evaluated on a functional level using PASS and FIST, as well as a participation level using BI. No participants withdrew from this program. Although this intervention training significantly improved trunk control capacity and everyday activities in stroke patients at home, the extent to which its effects can be generalized is limited. The participants were unable to perform this intervention alone due to safety concerns and the need to set up equipment. Furthermore, the post-test was administered immediately following completion of the 4-week program, restricting our capacity to determine retention of the intervention’s benefits. Additional research, including functional assessment throughout the follow-up period, would reveal the long-term efficacy of the training program used in this study.

## Conclusion

This study demonstrates that home-based training utilizing a seated balance disc combined with input from a smartphone inclinometer may increase trunk control and daily activity in individuals recovering from stroke. One benefit of this training regimen is its ease of use, as a smartphone can be used to provide visual feedback and facilitate regain of trunk control and movement. In addition to conventional physical therapy, this intervention provides safe and convenient home exercises to improve and progress the rehabilitation of stroke patients.

## Supporting information

S1 Checklist CONSORT 2010 checklist of information to include when reporting a randomised trial*. (DOC)

S1 Dataset (XLSX)

S1 File (PDF)
