# Supplementary material for: Home-based exercise using balance disc and smartphone inclinometer application improves balance and activity of daily living in individuals with stroke: A randomized controlled trial
Source: PLoS One. 2022 Nov 21;17(11):e0277870. doi: 10.1371/journal.pone.0277870 (PMC9678269; doi:10.1371/journal.pone.0277870)
Supplement: S1 File — (PDF) [file pone.0277870.s003.pdf]

Thesis Proposal

(English title): EFFECTS OF HOME-BASED EXERCISE USING BALANCE DISC WITH SMARTPHONE INCLINOMETER APPLICATION FEEDBACK ON SITTING BALANCE AND ACTIVITY OF DAILY LIVING IN INDIVIDUAL WITH STROKE

(ชื่อเรื่องภาษาไทย): ผลของการออกกำลังกายที่บ้านโดยใช้แผ่นฝึกการทรงตัวร่วมกับการให้ข้อมูลป้อนกลับโดยโปรแกรมมาตรวัดความเอียงในสมาร์ทโฟนต่อความสามารถในการทรงตัวในท่านั่งและการทำกิจวัตรประจำวันในผู้ป่วยโรคหลอดเลือดสมอง

Pantawit Aphiphaksakul

Akkradate Siriphorn

CHAPTER I

INTRODUCTION

1.1 Background and rationale

Stroke is a neurological disorder that has a massive impact globally. It causes brain damage and is associated with dysfunction of movement(1). Stroke was the second cause of death in 2015, and worldwide deaths had reached 6.3 million(2). Stroke is the third cause of death in Thailand, and Thailand has 250,000 new cases each year(3, 4). Stroke caused long-term disability in most cases(5) because it impaired many processes in the body, such as muscle weakness, deprivation of proprioception, and loss of feeling(6). It affects physical disability, such as sitting, standing, and walking, restricting other activities(7). Moreover, stroke survivors stay in bed when they have difficult mobility and physical activity in their lifestyle(8).

Stroke rehabilitation is a significant element in treating stroke patients that promotes brain activation and mobility by physical exercise. A recovery has high rate in first to 3 month and improve extend to 6 months. This period is important for training and increase activity for life(9). Previously studied recommended exercise improved muscle strength, cognitive abilities, balance, and mobility for stroke survivors(10). Physical exercise reduced the risk factor for subsequent stroke dysfunction. Training increases the efficiency of movement in terms of quality of living, but people with stroke have issues with disparity(11). Stroke rehabilitation can immediately start after the patient is medically stabilized in intensive care environments, i.e., the stroke unit(12). In Thailand, stroke survivors had an average length of stay in stroke units of 3 days, and the cost of treatment in a stroke unit was 10,206 Baths(13). Timing and intensity of training for recovery in stroke survivors limit at hospital and it led to sufficiency for recovery. Because stroke survivors must spend time for a recovery and improve function for activity for life extremely but length of stay in hospital is short period and intensity may not enough for recovery. Rehabilitation treatment for all stroke patients is recommended for three hours a day with intensive task-specific training, five days a week in inpatient facilities(12). Previous studies found an average length of hospital stay of 20 days for stroke survivors(14). After discharge from the hospital, the large proportion of stroke patients will need ongoing outpatient rehabilitation and additional long-term community rehabilitation for several months or years later(10, 15, 16). Several community-based rehabilitation programs were investigated, including home-based virtual reality training(17), robotic glove(18) and telerehabilitation(19). However, most home-based rehabilitation programs have used expensive equipment and are focused on the rehabilitation of hand functions(5, 16, 20, 21).

Sitting is essential for transfers and day-to-day activities(22). Stroke patients, who have trouble maintaining a sitting balance, display minimal progress in the transition to standing and often reducing walking recovery(23). Additionally, the spatio-temporal gait parameter in

individuals with chronic stroke was enhanced through additional trunk control training(23). Previous studies have also shown that trunk control training can improve sitting balance(24, 25). Several trunk control exercises can be used in stroke survivors, i.e., static sitting balance without back and arm support, dynamic sitting balance on a stable surface, and use of physio ball for dynamic training balance on an unstable surface(24, 25). The level of balance improvement, functional state, and ambulation is higher with additional trunk balancing exercises relative to conventional stroke exercises(26). A recent meta-analysis has shown that the implementation of trunk-based inpatient rehabilitation procedures provides short-term benefits to trunk efficiency and balance in stroke survivors(27). However, these exercises have been performed while the stroke survivors remain in the hospital, which may not be sufficient to improve long-term sitting balance and to improve other functional skills. To the best of our knowledge, only one study by Chan et al. (2014) explored task-related trunk training as a home-based program for stroke survivors. The program included five training sessions a week for six weeks. Each training session consists of 6 sets of movements, i.e., pelvic bridging, supine-to-sit, leaning in the forward-backward and lateral direction, turning the trunk while sitting, and reaching forward while sitting. The findings demonstrated a more significant increase in trunk muscle strength and trunk function in stroke patients(28) suggesting these task-related training could be used for trunk control training. However, no study has been investigated the effect of home-based task-specific training on trunk control.

Visual feedback seems to be another key factor in the success of balance training. Cho et al. (2012) showed that a 30-minute session, three days a week, for six weeks of visual feedback video game-based training, improved dynamic balance in stroke survivors(21). Likewise, Karasu et al. (2018) showed that the Nintendo Wii system as an adjunctive treatment increased the static and dynamic balance more than the control group(29). Interestingly, Hwang et al. (2017) found that a 30-minute session three days a week for four weeks of visual feedback training using force platform was better suited to optimizing static and dynamic balance than unstable surface training for stroke survivors(30).Thus, in this research, we believe that a combination of sitting balance training with unstable surface and real-time visual feedback using a simple mobile inclinometer application for home-based exercise would enhance the trunk control of stroke survivors after discharge.

1.2 Objectives of the study

- The study's objective is to investigate the effects of Effects of home-based exercise using balance disc with smartphone inclinometer application feedback on sitting balance and activity of daily living in individual with stroke.

1.3 Research question

- Does a home-based exercise using balance disc with smartphone inclinometer application feedback can improve sitting and activity of daily living balance in stroke survivors?

1.4 Hypothesis of the study

- Home-based sitting balance training using balance disc and the smartphone inclinometer feedback group would have substantially increased sitting balance relative to the control group at the end of the therapy session.

1.5 Keywords

- Sitting balance, Home-based exercise, Stroke, Activity of life

1.6 Scope of the study

- The venue of the study:. In the Bangkok metropolitan area.
- Population: Stroke survivors age 30 – 75 years old.
- Design of study:

  A single-blind randomized control study will be used to investigate the effects of home-based exercise using balance disc with smartphone inclinometer application feedback on sitting balance and activity of daily living to improve trunk control in stroke survivors.

1.7 Brief method

The researcher will screen stroke survivors who have agreed to enroll in the study. All participants who pass the screening with inclusion criteria will be evaluated in terms of demographic data. Participants will match by age, gender, BMI, and PASS score. Then, they will be randomly assigned to the experimental group and control group by random number in a concealed opaque envelope. The experimental group will receive a sitting balance training program for 30 minutes, five days per week for four weeks in addition of a conventional home rehabilitation program therapy for 30 minutes per day. The control group will receive conventional rehabilitation program therapy for 60 minutes per day. All outcomes measurements will be done two times at home that after discharge 1 week (pre-test) and the end of the training phase (post-test). Every week the researcher will visit participants for recheck program and equipment.

1.8 **Conceptual framework**

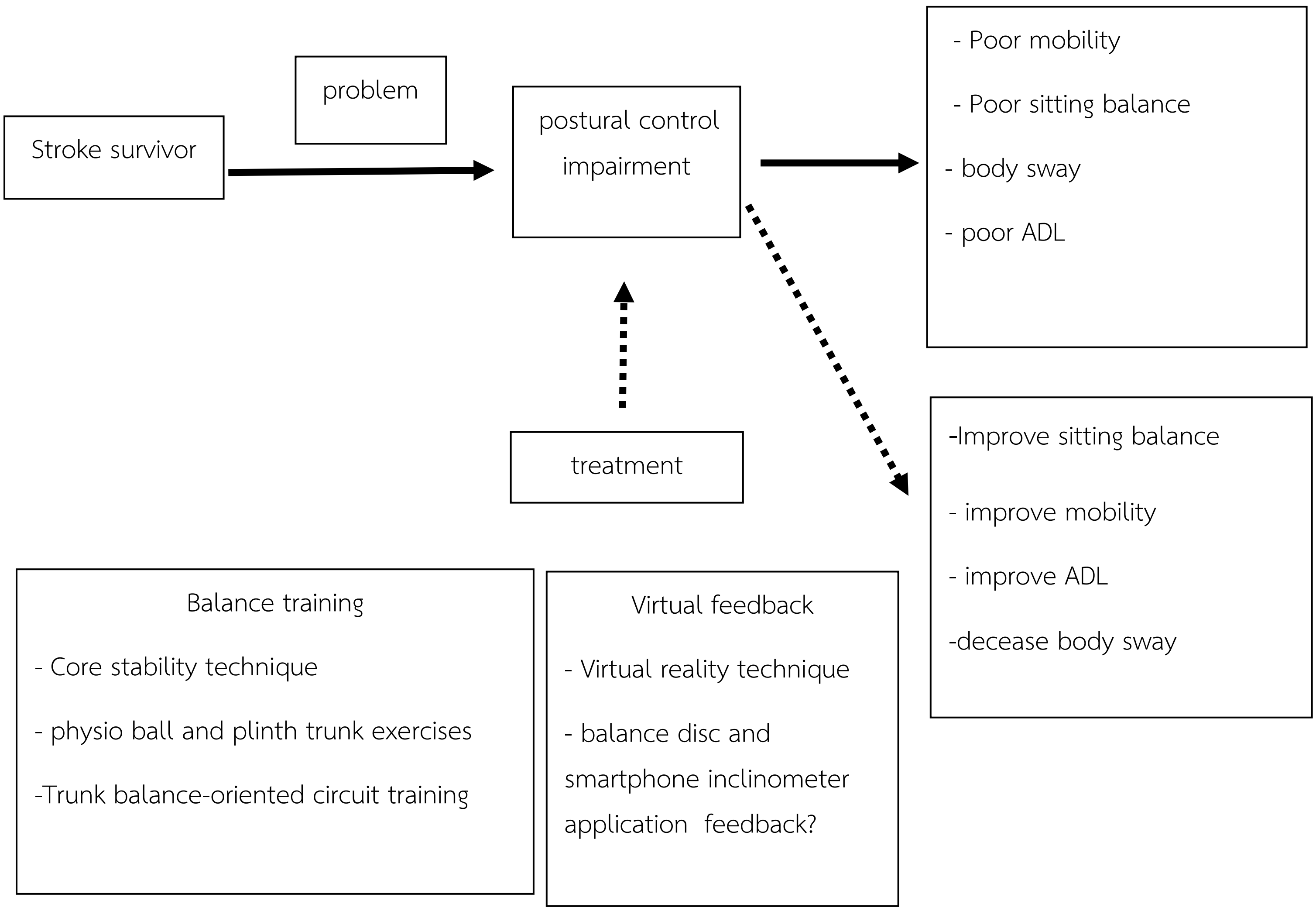

1.9 Expected benefits and applications.

- The results of the study will provide basic information for sitting balance training with balance disc and smartphone application at home.

CHAPTER II

REVIEW LITERATURE

1. Definition of stroke

Stroke is a neurological condition that occurs when brain-damaged such as infarction or hemorrhage by block or bleed in vascular. Brain, retina, or spinal cord loss function when oxygen inadequate. After the brain occurs cell death linked to the central nervous system (CNS) dysfunction(1). There are two main types of strokes: ischemic stroke (cerebral, retinal, and spinal infarction) and hemorrhagic stroke (intracerebral hemorrhage and subarachnoid hemorrhage). A stroke subtype includes anterior circulation syndrome, incomplete anterior circulation syndrome, lacunar syndrome, and posterior circulation syndrome(31).

2. Prevalence of stroke

Stroke is a significant cause of neurological disease. It is a leading cause of long term disability and death in stroke survivors(1). In 2016 there were 80.1 million people of stroke disease by classified ischemic stroke were 67.6 million, and hemorrhagic stroke was 15.3 million. There were 5.5 million deaths worldwide. Every year has a new stroke case 795, 000 people in ischemic and hemorrhagic, approximately 610 000 of first attacks, and 185,000 are recurrent stroke and occurred in low and middle-income countries(32). Thailand found 122 per 100,000 of the population of stroke survivors and has 7% of death(33).

3. Sign and symptoms

Stroke is a neurological disease. It has specific symptoms such as muscle weakness, loss of sensation, lack of proprioception, and loss of balance(31). The case of symptoms is the central nervous system (CNS) reduced activity. It is leading to abnormal function after stroke. The impairment of stroke can divide negative and positive symptoms(34). Moreover, another study categorized upper motor neuron impairment after stroke has three types, such as positive, negative, and adaptive types (35). In a positive type of muscle, overactivity is a velocity-dependent increase in the tonic stretch reflex. This symptom occurs Hyperreflexia, Clonus, Positive Babinski, Flexor spasms, and spasticity. It a positive sign abnormal movement and hyperexcitability of the stretch reflex stimulated tendon jerks(36). In negative type or muscle, under-activity occurs muscle weakness, impaired motor control, fatigue, and loss of Dexterity(34). Weakness is a significant problem because of muscle loss recruitment and firing rates in motor units, and the central nervous system(CNS) decrease function(35). Muscle under-activity lead to uncontrol balance and limit activity daily life in stroke survivor(37). Furthermore, muscle overactivity leads to muscle contracture, immobilization

joints, and poor balance. Both cause refer to abnormal movement after stroke(16). Balance in positive and negative symptoms are a significant cause of disability in a stroke survivor. They cannot control the static and dynamic position. It limits mobility and transfers to activity daily life(37).

4. Motor learning and training program in stroke survivor

After stroke have poor motor function and task in daily of life because brain decrease signal for control of movement that leading to poor skill and performance in stroke survivors. Training and timing are important for stimulate function and movement that brain get learn and improve skill. Motor learning have two components, i.e., explicit, and implicit motor learning. Explicit motor learning is ability of recall skill that it requires attention and remember of knowledge for skill. Implicit motor learning is giving signal for stimulate repeatedly and increase response for movement. Relationship between environment and person is recognizing together because movement is appropriate function for response task in daily of life. Learning task can preformed automatically without attention that is habituation, it develops from repetition of training or practice in stroke survivor. Process of development have 4 stages are encoding, consolidation, storage, and retrieval(38). Key factor of training is divided into 3 components, i.e., knowledge, cognitive skill, and context. Knowledge is ability to demand situation and self-generated response for task and movement. Cognitive skill is ability to analysis and solve problem when occur in task. Context is the relationship person and environment that can problem-solving, and decision-making for suitable movement for each the environment(39).

Intensity of training for improve movement need to be enough to stimulate brain function. Recommendation for stroke rehabilitation should start as soon as possible after stable medical conditions. Exercise for stroke have various components, i.e., aerobic, strength, flexibility, neuromuscular, balance, and gait training(10). Exercise and activity in stroke survivors should be set at modulate intensity, which could improve physical activity, cardiovascular, and reduce a risk sedentary factor(11). Task specific training must be set at high intensity for recovery of motor function and increase performance. Capacity for balance training and conventional therapy is range from 30 minutes to 1 hour, 2 to 5 days a week, and 2 to 8 weeks(40). However, training program can be adjusted by increased duration or decreased the frequency to suitable for each individual(40).

5. Concept of Postural control

Postural control is maintaining, achieving, or restoring a body on the base of support. Bodyweight refers to the point on the center of gravity and has a line of gravity pass body on the base of support(41). Postural control consists of seven components, i.e., musculoskeletal component, neuromuscular synergy, individual sensory system, sensory

strategy, internal representation, anticipatory mechanism, and adaptive mechanism. All of the component effect of maintaining and stable body posture (42). Sitting, standing, and walking are the primary position after stroke. This position must be training and learning patterns because many cases in stroke survivors have poor balance and limit of multidirectional movement. Musculoskeletal component effect stability position in stroke when trunk muscle, upper limb, and lower limb are weaknesses. It out of control body in the base of support(43). Loss of sensation is a significant cause of loss of balance because the information is lost, and sensory impairment does not include input data to the brain. Also, this case shows abnormal balance and movement after stroke(44). In sitting balance, use sensory such as somatosensory system, visual system, and vestibular system for balance position on the surface(45). Balance in-person use feedback somatosensory (70%), vision (10%), and vestibular (20%) in balance position on the surface(46). When one of the systems lose function, that occurs sway posture. However, the brain can integrate the sensory system for equilibrium posture(47). The internal representation is a relationship between body or peripheral part, and the environment shows in the brain that has coordination together. The brain can plan movement before doing real movement because maintaining a position for doing any movement prevent falling and stable posture and leading to prepare the muscle for maintaining position or perturbation that it is the anticipatory mechanism(48). The adaptive mechanism is a prevention system for unexpected perturbation and plans to maintain posture. The perturbation can divide into internal perturbation and external perturbation. The internal perturbation occurs when executing a self-generated force for movement such as reaching arm, transfer, and leaning forward that It perturbs balance in a stroke survivor. Furthermore, the external perturbation is an unexpected force from the outside body that it disturbed a balance and position. The body has feedback systems responses to external force with activating a coordinated of ankles, hips, and trunk strategies(48). Stroke survivors have a problem in motor and sensory strategies for postural control; it led to asymmetry body position, anticipatory mechanism decrease response to adjust the position. Because the brain has an injury and its effect on the CNS system. The performance of strategies related to internal and external risk factors that linked movement patterns and the ability to maintain the body in an equilibrium position. Loss of balance-related between disorders and functional impairment. Also, it must process complex systems together and activate coordination between the central nervous system and the peripheral system (46).

6. Balance and postural disorders in a stroke survivor

Balance and postural disorders are the leading cause to limit function and movement that cause of many components. It has a musculoskeletal component, neuromuscular synergies, individual sensory system, sensory strategies, internal representation, anticipatory

mechanism, and adaptive mechanism(46). A stroke survivor who has muscle weakness leading to an independent body and limb part in stability position(24). When asymmetrical body weight-bearing in a sitting position that limits multidirectional movement such as forward, backward, lateral side, and it affected to essential function in the quality of life(22). Trunk control is essential for maintaining and stabilizing an upright position that supports changing weight shifts in the sitting balance. It helps a control trunk for moving the arms and legs to assist in movement(25). Stroke survivors suffered an imbalance in a sitting position because of motor and sensory impairment. Most case lean-to affects side and sway body that increases the high risk of falling(49). When body shift weight to forward, affected side, and push away on affected side defined pusher syndrome. This cause stroke survivor cannot control upright position in sitting balance because of its impaired sensation of vertical(50, 51). Balance abnormality in stroke survivor involve brain damage and loss of system such as loss of sensory reweighting, perception impairment and loss of the internal representation system is pusher syndrome(47, 50, 51). This problem impact trunk control and performance of balance in a stroke survivor. Loss of balance control can be restored by rehabilitation. Brain plasticity improves motor and sensory function in stroke survivors. Motor learning and training activate neural in the central nervous system to stimulate neuroplasticity, and reboot system for functional recovery in the brain (15, 38). Sitting balance in stroke survivors disturbs by a disorder that limits function and delays recovery. It is an essential task for training because it improves trunk muscle and sensation. Stroke survivors have perception and learning problems in the initial stage(38). It links to the ability of shift weight body in sitting balance and doing every task in life such as reach arm for taking objects, eating, transfer, grooming prepares to stand, and to walk(22, 43).

7. Balance training for a stroke survivor

Various studies in trunk control have different training that is leading to increasing performance of sitting balance in stroke survivors (52). In 2019, Shin et al. designed the training for sitting balance exercises that use smartphones with task training. The result for this studied that it improves the ability of trunk control and gait in stroke survivors(23). In 2017, Verma et al. designed the Nintendo Wii balance board for balance exercises and used a game for varying tasks when training. This studied solve body sway, defined as the body, cannot adjust the center of mass in the gravitational environment. Moreover, games designed specifically for stroke survivor that it stimulates motor and sensory function and increase motivation while training(53). Exercise is leading to improve another function and solve body sway after stroke, increase weight-bearing, muscle strength, and postural control(22). Balance training is essential for the plan in the early phase after a stroke that measures the ability of mobility, standing, and gait. It increases recovery and motor relearning in stroke survivors. Trunk control and sitting balance is base for functional

movement, necessary to dynamic balance and mobility (43). Sitting is linked to the ability to reach for objects in multidirectional settings and lean body and arm length as individuals with stroke in daily life (54). It has been reported that sitting balance is essential to assessment and training in hospital and after discharge for stroke survivors (55). Previous research reported activation of proprioceptive sense strengthens the regulation of the trunk and stabilizes the body by proprioceptive neuromuscular Facilitation procedure, which relates to practical motions in everyday life(56). Furthermore, Fukata showed that sitting performance is effectively improved by sitting training by tilt surface. It stimulates perceptive sensation and increases somatosensory information in the femur and hip on the least affected side(57). There is a study that used an unstable surface for trunk training exercise; it is a benefit for the performance of trunk control in stroke survivors (58).Thus, balance exercise is important for add-in programs and planning at the hospital and after discharge. Nevertheless, the influence of balance exercises at home is still not evidenced enough for the program. Home program training is essential for stroke survivors after discharge.

8. Balance assessment

Balance assessment is important to evaluate the performance of trunk control, and it is leading to the prediction of an essential function in daily life (59). It uses for screening and classifying stroke patients who have balance impairment. Thus, the user must select an appropriate measurement. Balance assessment can divide into two categories, which are laboratory tests and clinical tests. In this session, focus sitting balance measurement and activity daily of life and performance of trunk control.

8.1 Clinical balance tests

The clinical tests are proper measurements for rehabilitation in hospitals and homes. It has various tests that easy to evaluate balance impairment in stroke survivors.

8.1.1 Function in sitting test (FIST)

Function in the sitting test (FIST) is a measurement that assesses sitting balance ability is a good predictor of recovery in stroke survivors. It assesses the performance of sitting balance and basic function in sitting position that shows the performance of trunk balance and stability(60, 61). This measurement consists of 14 item which has a total score of fifty-six points. A five-point scale ranging from 0-4 points is used. Zero is a complete assistance, and four is Independent. It takes 15 minutes to access. The FIST has high concurrent validity as compared to the BBS and FIM (r= .851, r= .712), have a standard error of measurement (SEM) was 1.40, have minimal detectable change (MDC) of 5.5 points. FIST score change ≥6.5 points determining the MCID have large effect size (.83), standardized response mean was

1.04, and index of responsiveness was 1.07 (52). The S-FIST has high internal consistency in acute stroke patients (Cronbach's α-coefficient = 0.97) (62).

8.1.2 Postural assessment scale for stroke patient (PASS)

The postural assessment scale for stroke patients has 12 items performance-based scale use for assessing and monitoring postural control in a stroke survivor. It divides two parts, such as maintaining a posture have five sections, and change a posture has seven sections. A four-point scale ranging from 0-3 points is used. This measurement has a cut-off score of 3.5 points for static PASS (sensitivity 77.9%; specificity 82.1%),8.5 points for dynamic PASS (sensitivity 77.9%; specificity 82.5%), and 12.5 points for total PASS (sensitivity 78.9%; specificity 83.7%)(54). It has good construct validity that high correlation with Functional Independence Measure (FIM) (r=0.73), showed high interrater and with an instrumental measure of postural stabilization(r=0.48). Moreover, this tool has high internal consistency (Cronbachαcoefficient=0.95)(63).

8.1.3 Barthel index (BI)

Barthel index (BI) evaluates disability. Inpatient and outpatient stroke suffered a problem in essential function movement in daily life. This tool assesses for the performance of stroke patients when setting a goal and program in rehabilitation.it is a predictor of the process in training that affects their activity of life. This measurement consists of 10 activities daily of life. It has intraclass correlation coefficient (ICC) ≥ 0.83, The BI and FIM motor subscale showed high internal consistency (α coefficient ≥ 0.84), high concurrent validity (Spearman's correlation coefficient r > 0.92(56). Moreover, it has moderate to excellent between raters for individual items (kappa value range, 0.53-0.94) and the total score (ICC = 0.94) (64).Sitting balance measurement is not a rigid pattern when evaluating stroke patients. It is essential in goal and planning for rehabilitation. This tool show performance of sitting balance and functional recovery in stroke survivors. It is leading to treatment and training suitable for individuals with stroke. Postural assessment scale for stroke patient (PASS) evaluates basic mobility and change position, Function in sitting test (FIST) showed performance sitting balance with task and Barthel index (BI) assess overall activity daily of life in a stroke patient. It helps to find problem impairment and process step by step that easies to manipulation.

CHAPTER III

MATERIAL AND METHODS

3.1 Research design

A single-blind randomized control study will be used to investigate the effects of home-based exercise using balance disc with smartphone inclinometer application feedback on sitting balance and activity of daily living to improve trunk control in stroke survivors. The researcher will screen stroke survivors who have agreed to enroll in the study. All participants who pass the screening with inclusion criteria will be evaluated in terms of demographic data. Participants will match by age, gender, BMI, and PASS score. Then, they will be randomly assigned to the experimental group and control group by random number in a concealed opaque envelope. The experimental group will receive a sitting balance training program for 30 minutes, five days per week for four weeks in addition of a conventional home rehabilitation program therapy for 30 minutes per day. The control group will receive conventional rehabilitation program therapy for 60 minutes per day. All outcomes measurements will be done two times at home that after discharge 1 week (pre-test) and the end of the training phase (post-test). Every week the researcher will visit participants for recheck program and equipment.

3.2 Sample size

The sample size was calculated using version 3.1.9.7 of the G*Power program based on the effect size of previous study(28). The result of the sample size estimation when setting power at 80 percent, alpha at 0.05, and the effect size at 0.48 was 28 participants (14 per group) (Figure 1). The drop-out rate was set at 10%, requiring a minimum of 32 participants (16 per group).

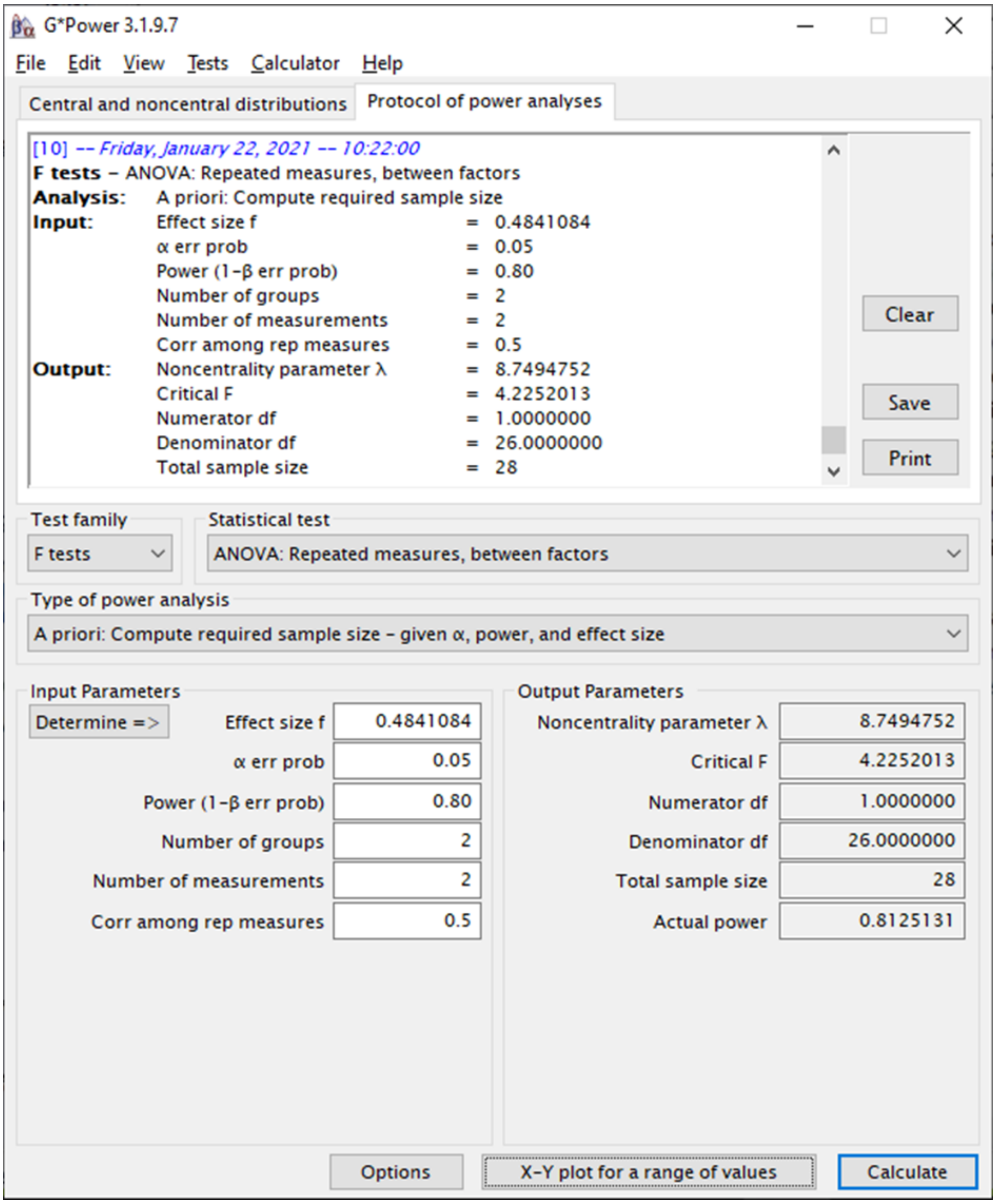

Figure 1. Sample size calculation. The calculated total sample size was 28 participants.

With the dropout rate at 10%, the total sample size will be 32 participants.

3.3 Participants

Thirty-two stroke survivors aged between 30 and 75 years will be recruited direct contact with physic therapists who take care of patients with a stroke at home. This requires permission from caring physical therapists and family members of the participants and from all 50 districts of Bangkok. And who pass the screening with inclusion and exclusion criteria will be recruited for this study. All participants will be informed about the test procedure and the training protocol. The inclusion and exclusion criteria for recruiting of participants are as follows:

Inclusion criteria

1. Diagnosis of the first stroke resulting in hemiplegia in the past three months.
2. Discharge from hospital and live at home.
3. Aged between 30-75 years old.
4. Modified Rankin scale ≥3
5. Can sitting independently without support.
6. Have a PASS score ≤ 12.5 points.
7. Have caregiver could get support for the home-based program.
8. No vision issues that cannot be fixed by glasses or contact lenses.
9. No history of back surgery, scoliosis, and current low back that impact on sitting performance.
10. can communication and able to follow the command.

Exclusion criteria

1. Have other neurological conditions, such as Parkinson’s disease, cerebellar disorder.
2. Have uncontrolled hypertension.
3. Have unstable sign or accident during study.
4. Unable to follow a command.
5. Miss Program more than 4 sessions out of 20 sessions
6. Participants are not willing to continue their research.
7. The participants had taken more than 4 consecutive sessions breaks.

In the preparation process, the researcher will explain the objectives, study process and the benefits of this research. The participants will then be requested to provide written informed consent to participate in this study. The ratio of ages 30-75 years will be stratified sampling divided into 5 stages(30-39 years,40-49 years, 50-59 years, 60-69 years,70-75 years).The genders will be recruited from stratified sampling. For the data collection, the researchers will enter the code of the volunteers anonymously in the participant record form. The participant's data will be kept confidential and limited to those who can access only the research team. The data will be recorded and destroyed after that period. The researcher will report general information.

3.4 Instrumentations

The following instrumentations will be used in this study:

3.4.1 Balanced disc

3.4.2 A chest strap

3.4.3 extended arm

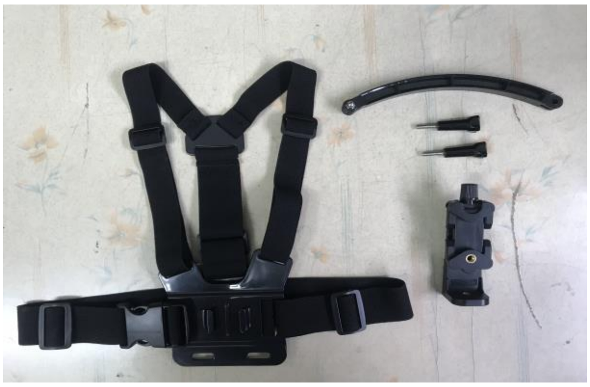

Figure 3. A chest strap and extended arm

3.4.4 Smartphone

3.4.5 The Chair (Chair size 30 * 47 cm^2.)

3.5 Research protocol

All participants will be screened with a questionnaire to collect participant Characteristics such as age, gender, weight, height, BMI, vision test, PASS score, FIST score, Barthel index and the eligibility checklist related to inclusion and exclusion criteria. The screening process will take place in the homes of the participants. It would make an appointment with a suitable date and time for the participants. The screening will be performed by a research assistant who is a licensed physical therapist who is not involved in intervention. This process will

take about 20 minutes. The researcher, who will not be involved in the assessment of the outcome, will teach an exercise program to participants at participant home. A leaflet of the exercise prescription will also be given to the participants. The caregiver will be well instructed to conduct the exercise intervention with the participants. In experimental group receive the equipment consists of a balanced disc, a chest strap with an extended arm, a smartphone, and the chair. This smartphone was installed with the Compass Inclinometer program(BrainLaboratories,availablefromhttps://play.google.com/store/apps/details?id=com.brainlab.tiltmeter) (Figure 6). The researcher will demonstrate the use of the devices and the training program. The inclinometer software is an Android smartphone application. The mobile will be fitted with a chest harness and an extension arm to place the smartphone on the chest of the participants. The application shows 3 inclinometers on the screen, each showing the degree of tilt of the mobile. When exercising, participants will be asked to take a look at the smartphone screen and move according to the exercise program.

To perform the exercise, the participant will assume the starting position (Figure 7), which is sitting on a balance disc on a chair and a foot flat on the floor. If the participant's feet cannot be on the floor, the researcher will use a step platform to support both feet. In order to avoid falling from the chair, the participants will be required to wear a belt around the waist all the time. The researcher will train the caregivers to maintain the safety of the participant during the exercise by sitting on the vulnerable side indenting the back of the participant and putting the caregiver's hand close to the belt. The exercise program has five levels as follows:

Level 1: Static sitting for 2 minutes with no movement or less than 5 degrees of change of tilting degree detected by the inclinometer.

Level 2: Lean forward, backward, lateral, and rotation of the trunk to both sides with a tilting degree detected by an inclinometer of about 15 degrees(65) move to the end point and hold 5 seconds then back to starting position, repeat 15 times per set, 3 sets of each direction;

Level 3: Lean forward, backward, lateral, and rotation of the trunk to both sides with a tilting degree detected by an inclinometer of about 30 degrees(65) move to the end point and hold 5 seconds then back to starting position, repeat 15 times per set, 3 sets of each direction;

Level 4: Lean forward, backward, lateral, and rotation of the trunk to both sides with a tilting degree detected by an inclinometer of about 45 degrees(65) move to the end point and hold 5 seconds then back to starting position, repeat 15 times per set, 3 sets of each direction;

Level 5: Lean forward, backward, lateral, and rotation of the trunk to both sides with a tilting degree detected by an inclinometer of about 60 degrees(65) move to the end point and hold 5 seconds then back to starting position, repeat 15 times per set, 3 sets of each direction.

All participants will start at level 1. If the participants can perform each level correctly in three consecutive sessions, the participant can move to the next level. Caregivers will be advised to verify the correctness of the exercise and to determine that the participant will pass the level or not. If the participants are unable to pass the level, they must resume their training at the same level until they can be achieved properly and fully. The experimental group will be trained in a sitting balance program for 30 minutes per day, five days a week, four weeks, in addition to the conventional home rehabilitation program they already received 30 minutes per day. The caregiver would be asked to provide support for the home-based program, particularly for participants' safety concerns at all times during the exercise program. Participants in the control group will be asked to continue the conventional home rehabilitation program for four weeks. The conventional program includes basic physical movements such as passive and active range of motion exercise in the upper and lower limbs on both sides, bed mobility training, and sitting balance training. This program is supposed to last 30 minutes.

Outcome measurements

All outcome assessments will be carried out on the basis of pre-test and post-test. All assessments of the outcome will be measured by a research assistant who is a certified physical therapist who is not aware of the participant group. The outcome measurements consist of Function in sitting test (FIST), Postural assessment scale stroke patient (PASS), and Barthel Index (BI).

6.1 Function in sitting test (FIST)

The function in the sitting test will be used to measure the clinical balance. It assesses the performance of the sitting balance, consisting of 14 items as follows: 1. Anterior nudge, 2. Posterior nudge, 3. Lateral nudge, 4. Static sitting, 5. Sitting, move head side to side (nod "no"), 6. Sitting, eyes closed, 7. Sitting, lift the foot, 8. Turn and pick up objects from behind in preferred direction, 9. Reach forward with outstretched hand at shoulder height, 10. Lateral reach with a hand at shoulder height, 11. Pick object up off floor, 12. Posterior scooting (5 cm.), 13. Anterior scooting (5 cm.), and 14. Lateral scooting(5 cm.). A five-point scale of 0-4 points is used (zero for complete assistance and four for independent assistance). The maximum FIST score is 56 (60).

6.2 Postural assessment scale in stroke patient (PASS)

The postural assessment scale of the stroke patient scale will be used as a clinical balance measurement tool. The PASS consists of twelve items as follows: 1. sitting without support, 2. standing with support, 3. standing without support, 4. standing on a non-paretic leg, 5. standing on a paretic leg, 6.supine to affected side lateral, 7.supine to non-affected side lateral, 8.supine to sitting up on the edge of the mat, 9. sitting on the edge of the mat to supine, 10.sitting to standing up, 11.standing up to sitting down, 12. standing, picking up a pencil from the floor) to evaluate posture change position(dynamic). A four-point scale ranging from 0-3 points is used (zero indicates the lowest level of function, and three indicates the highest level of function). A maximum score of PASS is 36 (69).

6.3 Barthel index (BI)

The Barthel index will be used to measure the clinical function of ADL. It measures the operation of everyday life consisting of the 10 ADL as follows: 1.feeding, 2.bathing, 3.grooming, 4.dressing, 5.bowels, 6.bladder, 7.toilet use, 8.transfer(bed to chair and back), 9. mobility(on level surfaces), 10.stairs. This tool has a three-point scale rating that is zero, implies that it is not effective, 5 = requires support, and 10 = autonomous. The highest Barthel index score is 100 (70).

COVOD-19 prevention

During the COVID-19 outbreak, the researcher made a phone call to query about the health symptoms of the participants and their families prior to their home visit. On the day of the visit, the researcher and participants will measure the temperature of the body, wear masks all the time, wash hands with alcohol gel before and after the visit and avoid excessive exposure.

3.6 Statistical analysis

Data will be analyzed using SPSS version 20.0 for static analysis, with a significant level set at $p < 0.05$. Descriptive statistics will be used to describe demographic and clinical data. All data will be presented as mean ±SD. The Shapiro-Wilk test will be used to determine the normal distribution of the data. The transformation of the logarithm will be performed for nonnormal-distributed parameter(s). Two-way mixed ANOVA (2 groups x 2 times) followed by Bonferroni posthoc test will be used to evaluate the change in the measurement of the results.

3.7 Ethical consideration

Participants will be explained objectives process benefits and the dangers that may occur, including unwanted side effects that may occur during research and while testing. However,

if adverse events occur from this research. The participant will be helped. If there is an event that is at risk of accident, the researcher will send the participant to a nearby hospital and coordinate with the agency for the subjects to see a doctor and perform service procedures quickly. The researcher will be responsible for all medical expenses. The subject's personal information will be kept confidential and restricted to those who can access only the researcher. The individual pictured in Figure 7 has provided written informed consent to publish their image alongside the manuscript.

3.8 Risk and the investigator's responsibility

Accident while exercise program is a potential risk. In the previous studies have not occurred. However, the researcher will be careful and follow all participation while in program. The researcher will visit every week for check equipment and participation. If the participant has accident in exercise program the researcher goes to participation home immediately and assessment before transfer participation to hospital.

3.9 Limitation in research

The study's weakness is the lack of equipment and the inability to exercise alone. It must be worn while training.

3.10 Gantt chart

	Feb 2020	Mar 2020	Apr 2020	May 2020	Jun 2020	Jul 2020	Aug 2020	Sep 2020	Oct 2020	Nov 2020	Dec 2020	Jan 2021	Feb 2021	Mar 2021	Apr 2021	May 2021	Jun 2021	Jul 2021	Aug 2021
Literature review	✓	✓	✓	✓	✓														
Write a thesis proposal	✓	✓	✓	✓	✓														
Defend thesis proposal					✓	✓	✓												
Ethical considerations								✓	✓	✓	✓	✓							
Data collection													✓	✓	✓	✓	✓		
Data analysis																✓	✓		
Writing and send a publication																	✓	✓	
Writing a thesis																	✓	✓	
Defend thesis																		✓	✓

3.11 Budget

Description	Total (THB)
**Equipment and Supplies**	
- Smartphone	3,000 * 14 = 42,000
- Arm extension camera	400 * 14 = 5,600
- Chest strap	150 * 14 = 2,100
- Balance disc	500 * 14 = 7,000
- A 4 paper	500
- Report price	500
- Travelling expense (5 months)	2,000 * 5 = 10,000
- The Chair	300 * 14 = 4,200
- Copy fee	2,000
**Total**	**73,900**

References

1. Coupland AP, Thapar A, Qureshi MI, Jenkins H, Davies AH. The definition of stroke. J R Soc Med. 2017;110(1):9-12.
2. Benjamin EJ, Virani SS, Callaway CW, Chamberlain AM, Chang AR, Cheng S, et al. Heart Disease and Stroke Statistics-2018 Update: A Report From the American Heart Association. Circulation. 2018;137(12):e67-e492.
3. Saengsuwan J, Suangpho P, Tiamkao S. Knowledge of Stroke Risk Factors and Warning Signs in Patients with Recurrent Stroke or Recurrent Transient Ischaemic Attack in Thailand. Neurol Res Int. 2017;2017:8215726.
4. Suwanwela NC. Stroke epidemiology in Thailand. J Stroke. 2014;16(1):1-7.
5. Dabrowska-Bender M, Milewska M, Golabek A, Duda-Zalewska A, Staniszewska A. The Impact of Ischemic Cerebral Stroke on the Quality of Life of Patients Based on Clinical, Social, and Psychoemotional Factors. J Stroke Cerebrovasc Dis. 2017;26(1):101-7.
6. Kessner SS, Bingel U, Thomalla G. Somatosensory deficits after stroke: a scoping review. Top Stroke Rehabil. 2016;23(2):136-46.
7. Morgan P. The relationship between sitting balance and mobility outcome in stroke. Australian Journal of Physiotherapy. 1994;40(2):91-6.
8. Danielsson A, Willen C, Sunnerhagen KS. Physical activity, ambulation, and motor impairment late after stroke. Stroke Res Treat. 2012;2012:818513.
9. Belagaje SR. Stroke Rehabilitation. Continuum (Minneap Minn). 2017;23(1, Cerebrovascular Disease):238-53.
10. Han P, Zhang W, Kang L, Ma Y, Fu L, Jia L, et al. Clinical Evidence of Exercise Benefits for Stroke. Adv Exp Med Biol. 2017;1000:131-51.
11. Billinger SA, Arena R, Bernhardt J, Eng JJ, Franklin BA, Johnson CM, et al. Physical activity and exercise recommendations for stroke survivors: a statement for healthcare professionals from the American Heart Association/American Stroke Association. Stroke. 2014;45(8):2532-53.
12. Hebert D, Lindsay MP, McIntyre A, Kirton A, Rumney PG, Bagg S, et al. Canadian stroke best practice recommendations: Stroke rehabilitation practice guidelines, update 2015. Int J Stroke. 2016;11(4):459-84.
13. Rajborirug K, Tumviriyakul H, Suwanno J. Effects of Stroke Unit Care in Acute Ischemic Stroke Patient Ineligible for Thrombolytic Treatment. J Med Assoc Thai. 2017;100(4):410-7.
14. Potluri R, Wasim M, Markandey B, Kapour A, Khouw N, Carter P, et al. Length of hospital stay is shorter in South Asian patients with ischaemic stroke. International Journal of Cardiology. 2015;187:190-1.
15. Hara Y. Brain Plasticity and Rehabilitation in Stroke Patients. Journal of Nippon Medical School. 2015;82(1):4-13.

16. Trompetto C, Marinelli L, Mori L, Pelosin E, Curra A, Molfetta L, et al. Pathophysiology of spasticity: implications for neurorehabilitation. Biomed Res Int. 2014;2014:354906.
17. Sheehy L, Taillon-Hobson A, Sveistrup H, Bilodeau M, Yang C, Welch V, et al. Home-based virtual reality training after discharge from hospital-based stroke rehabilitation: a parallel randomized feasibility trial. Trials. 2019;20(1):333.
18. Bernocchi P, Mulè C, Vanoglio F, Taveggia G, Luisa A, Scalvini S. Home-based hand rehabilitation with a robotic glove in hemiplegic patients after stroke: a pilot feasibility study. Topics in Stroke Rehabilitation. 2018;25(2):114-9.
19. Dodakian L, McKenzie AL, Le V, See J, Pearson-Fuhrhop K, Burke Quinlan E, et al. A Home-Based Telerehabilitation Program for Patients With Stroke. Neurorehabil Neural Repair. 2017;31(10-11):923-33.
20. Kumar D, Sinha N, Dutta A, Lahiri U. Virtual reality-based balance training system augmented with operant conditioning paradigm. Biomed Eng Online. 2019;18(1):90.
21. Cho KH, Lee KJ, Song CH. Virtual-reality balance training with a video-game system improves dynamic balance in chronic stroke patients. Tohoku J Exp Med. 2012;228(1):69-74.
22. Haruyama K, Kawakami M, Otsuka T. Effect of Core Stability Training on Trunk Function, Standing Balance, and Mobility in Stroke Patients. Neurorehabil Neural Repair. 2017;31(3):240-9.
23. Shin DC. Smartphone-based visual feedback trunk control training for gait ability in stroke patients: A single-blind randomized controlled trial. Technol Health Care. 2019.
24. Cabanas-Valdes R, Bagur-Calafat C, Girabent-Farres M, Caballero-Gomez FM, Hernandez-Valino M, Urrutia Cuchi G. The effect of additional core stability exercises on improving dynamic sitting balance and trunk control for subacute stroke patients: a randomized controlled trial. Clin Rehabil. 2016;30(10):1024-33.
25. Karthikbabu S, Nayak A, Vijayakumar K, Misri Z, Suresh B, Ganesan S, et al. Comparison of physio ball and plinth trunk exercises regimens on trunk control and functional balance in patients with acute stroke: a pilot randomized controlled trial. Clin Rehabil. 2011;25(8):709-19.
26. Buyukavci R, Sahin F, Sag S, Dogu B, Kuran B. The impact of additional trunk balance exercises on balance, functional condition and ambulation in early stroke patients: Randomized controlled trial. FTR - Turkiye Fiziksel Tip ve Rehabilitasyon Dergisi. 2016;62:248-56.
27. Souza DCB, de Sales Santos M, da Silva Ribeiro NM, Maldonado IL. Inpatient trunk exercises after recent stroke: An update meta-analysis of randomized controlled trials. NeuroRehabilitation. 2019;44(3):369-77.
28. Chan BK, Ng SS, Ng GY. A home-based program of transcutaneous electrical nerve stimulation and task-related trunk training improves trunk control in patients with stroke: a randomized controlled clinical trial. Neurorehabil Neural Repair. 2015;29(1):70-9.

29. Karasu AU, Batur EB, Karatas GK. Effectiveness of Wii-based rehabilitation in stroke: A randomized controlled study. J Rehabil Med. 2018;50(5):406-12.
30. Hwang H-s, Kim J-h, Choi B-r. Comparison of the effects of visual feedback training and unstable surface training on static and dynamic balance in patients with stroke. J Phys Ther Sci. 2017;29(10):1720-2.
31. Hankey GJ. Stroke. Lancet. 2017;389(10069):641-54.
32. Benjamin EJ, Muntner P, Alonso A, Bittencourt MS, Callaway CW, Carson AP, et al. Heart Disease and Stroke Statistics-2019 Update: A Report From the American Heart Association. Circulation. 2019;139(10):e56-e528.
33. Kongbunkiat K, Kasemsap N, Thepsuthammarat K, Tiamkao S, Sawanyawisuth K. National data on stroke outcomes in Thailand. J Clin Neurosci. 2015;22(3):493-7.
34. Emos MC, Agarwal S. Neuroanatomy, Upper Motor Neuron Lesion. StatPearls. Treasure Island (FL): StatPearls Publishing

StatPearls Publishing LLC.; 2020.
35. Shepherd RB. Exercise and training to optimize functional motor performance in stroke: driving neural reorganization? Neural Plast. 2001;8(1-2):121-9.
36. Segal M. Muscle Overactivity in the Upper Motor Neuron Syndrome: Pathophysiology. Phys Med Rehabil Clin N Am. 2018;29(3):427-36.
37. Tyson SF, Hanley M, Chillala J, Selley A, Tallis RC. Balance disability after stroke. Phys Ther. 2006;86(1):30-8.
38. Dahms C, Brodoehl S, Witte OW, Klingner CM. The importance of different learning stages for motor sequence learning after stroke. Hum Brain Mapp. 2020;41(1):270-86.
39. Krakauer JW. Motor learning: its relevance to stroke recovery and neurorehabilitation. Curr Opin Neurol. 2006;19(1):84-90.
40. Cabanas-Valdes R, Cuchi GU, Bagur-Calafat C. Trunk training exercises approaches for improving trunk performance and functional sitting balance in patients with stroke: a systematic review. NeuroRehabilitation. 2013;33(4):575-92.
41. Pollock AS, Durward BR, Rowe PJ, Paul JP. What is balance? Clin Rehabil. 2000;14(4):402-6.
42. Sibley KM, Beauchamp MK, Van Ooteghem K, Straus SE, Jaglal SB. Using the Systems Framework for Postural Control to Analyze the Components of Balance Evaluated in Standardized Balance Measures: A Scoping Review. Archives of Physical Medicine and Rehabilitation. 2015;96(1):122-32.e29.
43. Park J, Gong J, Yim J. Effects of a sitting boxing program on upper limb function, balance, gait, and quality of life in stroke patients. NeuroRehabilitation. 2017;40(1):77-86.
44. Yoon HS, Cha YJ, Sohn MK, You JSH. Effect of rehabilitation on the somatosensory evoked potentials and gait performance of hemiparetic stroke patients. Technology and

health care : official journal of the European Society for Engineering and Medicine. 2018;26(S1):145-50.
45. Boo J-A, Moon S-H, Lee S-M, Choi J-H, Park S-E. Effect of whole-body vibration exercise in a sitting position prior to therapy on muscle tone and upper extremity function in stroke patients. J Phys Ther Sci. 2016;28(2):558-62.
46. Horak FB. Postural orientation and equilibrium: what do we need to know about neural control of balance to prevent falls? Age Ageing. 2006;35 Suppl 2:ii7-ii11.
47. Bronstein AM. Chapter 4 - Multisensory integration in balance control. In: Furman JM, Lempert T, editors. Handbook of Clinical Neurology. 137: Elsevier; 2016. p. 57-66.
48. Mansfield A, Aqui A, Danells CJ, Knorr S, Centen A, DePaul VG, et al. Does perturbation-based balance training prevent falls among individuals with chronic stroke? A randomised controlled trial. BMJ Open. 2018;8(8):e021510-e.
49. Ryerson S, Byl NN, Brown DA, Wong RA, Hidler JM. Altered trunk position sense and its relation to balance functions in people post-stroke. J Neurol Phys Ther. 2008;32(1):14-20.
50. Yang YR, Chen YH, Chang HC, Chan RC, Wei SH, Wang RY. Effects of interactive visual feedback training on post-stroke pusher syndrome: a pilot randomized controlled study. Clin Rehabil. 2015;29(10):987-93.
51. Lafosse C, Kerckhofs E, Vereeck L, Troch M, Van Hoydonck G, Moeremans M, et al. Postural abnormalities and contraversive pushing following right hemisphere brain damage. Neuropsychol Rehabil. 2007;17(3):374-96.
52. Arienti C, Lazzarini SG, Pollock A, Negrini S. Rehabilitation interventions for improving balance following stroke: An overview of systematic reviews. PLoS One. 2019;14(7):e0219781.
53. Verma S, Kumar D, Kumawat A, Dutta A, Lahiri U. A Low-Cost Adaptive Balance Training Platform for Stroke Patients: A Usability Study. IEEE Trans Neural Syst Rehabil Eng. 2017;25(7):935-44.
54. De Luca A, Giannoni P, Vernetti H, Capra C, Lentino C, Checchia GA, et al. Training the Unimpaired Arm Improves the Motion of the Impaired Arm and the Sitting Balance in Chronic Stroke Survivors. IEEE Trans Neural Syst Rehabil Eng. 2017;25(7):873-82.
55. Cannell J, Jovic E, Rathjen A, Lane K, Tyson AM, Callisaya ML, et al. The efficacy of interactive, motion capture-based rehabilitation on functional outcomes in an inpatient stroke population: a randomized controlled trial. Clin Rehabil. 2018;32(2):191-200.
56. Hwangbo PN, Don Kim K. Effects of proprioceptive neuromuscular facilitation neck pattern exercise on the ability to control the trunk and maintain balance in chronic stroke patients. J Phys Ther Sci. 2016;28(3):850-3.
57. Fukata K, Amimoto K, Inoue M, Sekine D, Inoue M, Fujino Y, et al. Effects of diagonally aligned sitting training with a tilted surface on sitting balance for low sitting performance in the early phase after stroke: a randomised controlled trial. Disabil Rehabil. 2019:1-9.

58. Haruyama K, Kasai K, Makino R, Hoshi F, Nishihara K. Quantification of trunk segmental coordination and head stability in laterally unstable sitting identifies aging and cerebellar ataxia. Clin Biomech (Bristol, Avon). 2019;63:127-33.
59. Fil Balkan A, Salci Y, Keklicek H, Cetin B, Adin RM, Armutlu K. The trunk control: Which scale is the best in very acute stroke patients? Top Stroke Rehabil. 2019;26(5):359-65.
60. Gorman SL, Radtka S, Melnick ME, Abrams GM, Byl NN. Development and validation of the Function In Sitting Test in adults with acute stroke. J Neurol Phys Ther. 2010;34(3):150-60.
61. Hashimoto K, Higuchi K, Nakayama Y, Abo M. Ability for basic movement as an early predictor of functioning related to activities of daily living in stroke patients. Neurorehabil Neural Repair. 2007;21(4):353-7.
62. Cabanas-Valdés R, Bagur-Calafat C, Caballero-Gómez FM, Cervera-Cuenca C, Moya-Valdés R, Rodríguez-Rubio PR, et al. Validation and reliability of the Spanish version of the Function in Sitting Test (S-FIST) to assess sitting balance in subacute post-stroke adult patients. Top Stroke Rehabil. 2017;24(6):472-8.
63. Benaim C, Pérennou DA, Villy J, Rousseaux M, Pelissier JY. Validation of a standardized assessment of postural control in stroke patients: the Postural Assessment Scale for Stroke Patients (PASS). Stroke. 1999;30(9):1862-8.
64. Hsueh IP, Lin JH, Jeng JS, Hsieh CL. Comparison of the psychometric characteristics of the functional independence measure, 5 item Barthel index, and 10 item Barthel index in patients with stroke. J Neurol Neurosurg Psychiatry. 2002;73(2):188-90.
65. Broderick JP, Adeoye O, Elm J. Evolution of the Modified Rankin Scale and Its Use in Future Stroke Trials. Stroke. 2017;48(7):2007-12.
66. Perera C, Chakrabarti R, Islam FMA, Crowston J. The Eye Phone Study: reliability and accuracy of assessing Snellen visual acuity using smartphone technology. Eye (Lond). 2015;29(7):888-94.
67. Bailey IL, Lovie-Kitchin JE. Visual acuity testing. From the laboratory to the clinic. Vision Res. 2013;90:2-9.
68. Preuss RA, Popovic MR. Quantitative analysis of the limits of stability in sitting. J Appl Biomech. 2010;26(3):265-72.
69. Huang YC, Wang WT, Liou TH, Liao CD, Lin LF, Huang SW. Postural Assessment Scale for Stroke Patients Scores as a predictor of stroke patient ambulation at discharge from the rehabilitation ward. J Rehabil Med. 2016;48(3):259-64.
70. Mahoney FI, Barthel DW. FUNCTIONAL EVALUATION: THE BARTHEL INDEX. Md State Med J. 1965;14:61-5.
